# Mad honey disease: A challenging encounter in the emergency department

**DOI:** 10.1002/ccr3.9555

**Published:** 2024-11-01

**Authors:** Sajjad Ahmed Khan, Anish Luitel, Rasmita Poudel, Bijita Aryal, Vivek K. Rauniyar, Surya Bahadur Parajuli

**Affiliations:** ^1^ Birat Medical College Teaching Hospital Morang Nepal; ^2^ Department of Clinical Neurology Birat Medical College Teaching Hospital Morang Nepal; ^3^ Department of Community Medicine Birat Medical College Teaching Hospital Morang Nepal

**Keywords:** alcohol interaction, grayanotoxins, hypotension, mad honey disease, syncope

## Abstract

Be aware of potential severe interactions between aged honey containing grayanotoxins and alcohol. Such combinations can exacerbate symptoms like syncope and hypotension, especially in elderly patients with pre‐existing conditions, necessitating careful evaluation and management.

## 1 INTRODUCTION

Syncope, characterized by a transient loss of consciousness due to a temporary reduction in cerebral perfusion, poses a significant diagnostic challenge, particularly in the elderly population. The differential diagnosis for syncope includes a range of cardiovascular, neurological, and metabolic disorders.^1^ Among these, alcohol consumption is a known contributor to syncope, with its effects on blood pressure and heart rate being well‐documented.^2^ However, the interaction of alcohol with other substances, such as honey, introduces additional complexities that can significantly alter clinical presentation.

One such complexity is the phenomenon known as “mad honey disease,” which arises from the consumption of honey contaminated with grayanotoxins, primarily found in nectar from rhododendron species.^3^ In regions where such honey is common, its consumption can lead to a range of symptoms including dizziness, syncope, hypotension, and bradycardia.^4^ The grayanotoxins in “mad honey” interfere with sodium channels in the body, leading to a decrease in blood pressure and heart rate, which may contribute to syncope and other systemic effects.^5^

In the context of the case at hand, the patient ingested a mixture of locally available alcohol and honey that had been stored for 2 years. This aged honey could potentially contain higher concentrations of grayanotoxins, thereby increasing the risk of “mad honey disease” symptoms.^6^ Coupled with his existing hypertension, this combination might have exacerbated his condition, leading to syncope and bilateral nasal bleeding. The presence of nasal bleeding, alongside dizziness and headache, further complicates the diagnosis, suggesting possible systemic effects or a coagulopathy.^7^

An additional diagnostic challenge emerged from the chest X‐ray, which revealed haziness in the bilateral lung fields, more pronounced in the left lung. This finding introduces a new layer of complexity in the context of “mad honey disease” and raises questions about potential pulmonary involvement or secondary effects of the toxic honey ingestion.

## 2 CASE HISTORY/EXAMINATION

A 60‐year‐old male of Rai ethnicity was referred to our emergency department from a community hospital. He presented with symptoms following an episode of syncope and a fall, which was subsequently accompanied by bilateral nasal bleeding. The nasal bleeding was described as fresh and scanty. Additionally, the patient reported headache, dizziness, and an occasional dry cough that had been persistent for the past 4 months. He had no accompanying symptoms such as fever, frothing from the mouth, vomiting, shortness of breath, chest pain, hemoptysis, hematemesis, melena, burning micturition, abnormal body movements, or jaundice.

The patient's medical history included a 12‐year history of hypertension, which was managed with medication. Recently, he had been advised by a local healer that a mixture of wild honey and alcohol could cure his chronic cough. Following this advice, he ingested approximately 400 mL of locally available alcohol mixed with 40 mL of honey that had been stored for 2 years. This mixture, which amounted to about 16 units of alcohol, was consumed the evening before his presentation to the hospital. He denied any history of smoking and had no other chronic conditions such as diabetes mellitus, thyroid disorders, epilepsy, tuberculosis, or chronic obstructive pulmonary disease (COPD). There was no prior history of surgical procedures.

On examination, the patient appeared ill but was fully alert and oriented, with a Glasgow Coma Scale (GCS) score of 15/15. His pupils were bilaterally reactive, and there were no signs of pallor, jaundice, clubbing, cyanosis, or edema. His hydration status appeared normal. Vital signs revealed hypotension with a blood pressure of 80/50 mmHg in the supine position and bradycardia with a pulse rate of 42 beats per minute. Notably, the patient had a regular respiratory rate but exhibited significant bradycardia and hypotension, which are classic signs of mad honey disease. All peripheral pulses were palpable, and there was no radio‐radial or radio‐femoral delay. The oxygen saturation was normal at 95%.

The chest examination was unremarkable, with clear heart sounds and no murmurs. However, the neurological examination revealed mild confusion and ataxia, which are symptoms sometimes associated with mad honey toxicity. The abdominal examination was soft and non‐tender, with no abnormal findings.

## 3 METHODS (DIFFERENTIAL DIAGNOSIS, INVESTIGATIONS, AND TREATMENT)

In addressing the patient's symptoms and history, several differential diagnoses were considered. Hypovolemic shock was a primary concern due to the possibility of bleeding, particularly in the context of the recent fall and the effects of alcohol. Alcohol‐induced hypotension and bradycardia were considered, given the patient's recent high intake of alcohol. However, the presence of bradycardia, hypotension, and confusion, alongside the use of potentially contaminated honey, suggested the possibility of “mad honey disease.” Head trauma from the fall was also a consideration, though no immediate signs of intracranial injury were observed. Nasal hemorrhage could be related to trauma or a possible coagulopathy. Infection was another potential cause, given the patient's history of cough and recent alcohol use, although systemic signs such as fever were absent.

**FIGURE 1:**
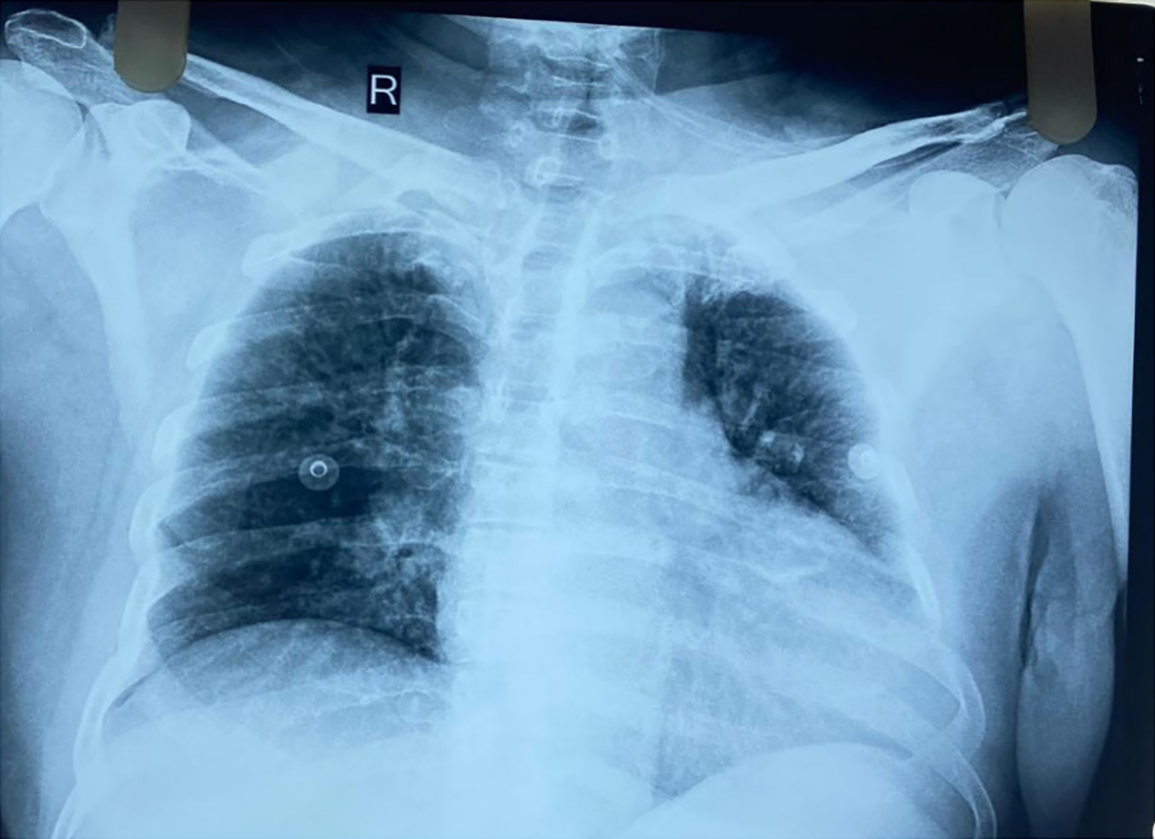
Chest X‐ray showing haziness in the bilateral lung fields, more pronounced in the left lung.

The chest X‐ray revealed haziness in the bilateral lung fields, more pronounced in the left lung (Figure 1). This finding prompted consideration of several additional factors. While haziness on X‐ray might initially suggest an infectious process, such as pneumonia or pulmonary edema, the context of mad honey disease and recent alcohol consumption necessitated exploration of less common pulmonary effects associated with toxic ingestion.

To evaluate these possibilities, a comprehensive diagnostic workup was initiated. Laboratory tests revealed a complete blood count (CBC) showing mild leukocytosis and normal hemoglobin levels, suggesting no significant acute infection or anemia. Renal function tests (RFT) showed normal creatinine and electrolyte levels, indicating no acute renal impairment. Liver function tests (LFT) were normal, ruling out acute liver dysfunction despite chronic alcohol use. Routine urine examination did not show significant abnormalities.

**FIGURE 2:**
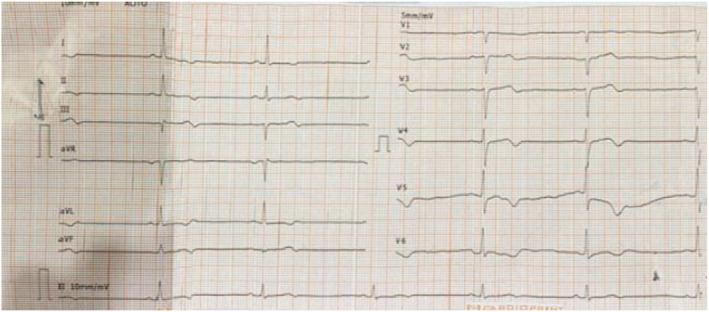
Electrocardiography (ECG) showing bradycardia consistent with the effects of grayanotoxin toxicity.

An electrocardiogram (ECG) revealed bradycardia with a heart rate of 42 beats per minute, consistent with the effects of grayanotoxin toxicity (Figure 2). A chest X‐ray showed bilateral lung haziness, particularly on the left, which was atypical for straightforward mad honey toxicity but warranted further evaluation. A CT scan of the head showed no intracranial hemorrhage or acute trauma. Echocardiography demonstrated normal cardiac function and structure.

Initial management focused on stabilizing the patient's condition and addressing the identified concerns. Fluid therapy was commenced with dextrose normal saline (DNS) to counteract hypotension. Vasopressors were administered to support blood pressure and ensure adequate perfusion. The patient was started on antibiotics, specifically ceftriaxone and sulbactam 1 gm IV twice daily for 5 days, to address potential infections. Pantoprazole was given for gastrointestinal protection, and ondansetron was administered to manage nausea. Given the patient's history of chronic alcohol consumption and suspected mad honey toxicity, thiamine was provided to prevent Wernicke's encephalopathy. Additionally, activated charcoal was administered as a precautionary measure to reduce the absorption of any residual grayanotoxins. A Foley catheter was placed for monitoring urine output and assessing kidney function. The patient was positioned with the head elevated to reduce intracranial pressure and prevent further complications.

## 4 RESULTS AND CONCLUSION (OUTCOME AND FOLLOW‐UP)

The immediate treatment resulted in stabilization of the patient's vital signs. The management approach effectively addressed the hypotension and provided symptomatic relief. The laboratory and imaging results confirmed no acute intracranial or renal complications. The patient's condition improved significantly with treatment, indicating a likely resolution of mad honey toxicity symptoms.

The haziness in the bilateral lung fields on the chest X‐ray, though not directly explained by “mad honey disease,” was monitored closely. It was managed as a potential secondary effect or complication, though no definitive pulmonary pathology was identified.

The patient was admitted to the ICU for close monitoring and continued management. Follow‐up care involved a thorough review of the diagnostic test results to refine the diagnosis and adjust the treatment plan as needed. Ongoing monitoring focused on assessing vital signs, evaluating the effectiveness of the treatment, and ensuring comprehensive care. The patient's response to initial therapies guided further management strategies and recovery efforts, with no signs of delayed effects from grayanotoxins observed. The haziness in the lung fields was resolved or improved with treatment, supporting a positive overall outcome.

## 5 DISCUSSION

This case presents an intriguing clinical scenario where an elderly male experienced syncope and bilateral nasal bleeding following the consumption of a mixture of aged honey and alcohol. The unusual combination of these substances highlights a rare interaction that is not commonly documented in medical literature, making this case particularly noteworthy.

Grayanotoxins, which are found in honey derived from the nectar of rhododendron species, are known to cause symptoms such as dizziness, hypotension, bradycardia, and gastrointestinal distress due to their interference with sodium channels.^3^ While the manifestations of “mad honey disease” are well‐documented, they are usually observed in isolation from alcohol consumption.^4^ This case introduces a novel aspect by examining how the combination of aged honey and alcohol might amplify or alter the clinical effects of grayanotoxins. The aged honey could have contained higher concentrations of grayanotoxins, potentially leading to a more severe presentation of symptoms. Additionally, alcohol's known vasodilatory effects and its impact on cardiovascular function might have exacerbated the hemodynamic disturbances caused by the grayanotoxins, leading to profound hypotension and bradycardia, and contributing to the observed syncope.

The finding of bilateral lung field haziness on the chest X‐ray adds another layer of complexity to this case. This pulmonary abnormality, especially more pronounced in the left lung, is not commonly associated with mad honey disease. It could indicate additional systemic effects or complications resulting from the combined ingestion of grayanotoxins and alcohol. Potential explanations for the haziness include aspiration, an inflammatory response, or toxic pulmonary edema, which could be aggravated by the patient's underlying health conditions or the effects of the substances ingested. While “mad honey disease” primarily affects the cardiovascular and gastrointestinal systems, this case underscores the potential for broader systemic effects, including respiratory involvement.

The interaction between alcohol and grayanotoxins may enhance or alter their individual effects, leading to a more severe clinical presentation. Alcohol can cause vasodilation and hypotension, which, when combined with the sodium channel effects of grayanotoxins, might result in greater hemodynamic instability.^8^ This interaction could also explain the patient's confusion and ataxia, as alcohol's impact on cognitive function and coordination might have compounded the effects of the grayanotoxins. The aged honey, potentially containing higher levels of toxins, further complicates the clinical picture.

This case emphasizes the importance of considering potential interactions between substances when diagnosing and managing complex cases. The findings suggest that the combined effects of aged honey and alcohol can lead to severe and multifaceted clinical outcomes that may not be immediately apparent from the classic presentation of mad honey disease alone. It highlights the need for healthcare providers to be vigilant about the possible dangers of combining unregulated substances and to be aware of the broader systemic effects that such combinations might produce.

Furthermore, this case underscores the need for increased awareness and research into the interactions between honey, particularly when contaminated with grayanotoxins, and alcohol. Understanding these interactions more comprehensively could lead to better diagnostic and management strategies, potentially preventing similar cases in the future. The additional finding of lung field haziness suggests that the systemic effects of such toxic combinations may extend beyond traditional presentations, warranting further investigation into their implications for clinical practice.

## 6 CONCLUSION

In conclusion, this case underscores the complex interplay between aged honey and alcohol in exacerbating the clinical manifestations of “mad honey disease.” The interaction between grayanotoxins and alcohol appears to produce a more severe and multifaceted presentation, including symptoms not typically associated with mad honey intoxication alone, such as bilateral nasal bleeding and pulmonary abnormalities. The additional finding of lung field haziness suggests broader systemic effects that warrant further investigation. This case highlights the critical need for heightened awareness among healthcare providers about the potential risks of combining unregulated substances and emphasizes the importance of ongoing research to better understand these interactions and their implications for diagnosis and treatment.

## AUTHOR CONTRIBUTIONS

**Sajjad Ahmed Khan:** Conceptualization; writing – original draft. **Anish Luitel:** Conceptualization; writing – original draft. **Rasmita Poudel:** Conceptualization; writing – original draft. **Bijita Aryal:** Writing – original draft. **Vivek K. Rauniyar:** Writing – review and editing. **Surya Bahadur Parajuli:** Writing – review and editing.

## FUNDING INFORMATION

None.

## CONFLICT OF INTEREST STATEMENT

The authors declare no conflicts of interest.

## CONSENT

Written informed consent was obtained from the patient to publish this report in accordance with the journal's patient consent policy.

## Data Availability

Data will be provided by the corresponding author upon reasonable request. Images uploaded in the separate files.
